# Cloaking In-Plane Elastic Waves with Swiss Rolls

**DOI:** 10.3390/ma13020449

**Published:** 2020-01-17

**Authors:** Younes Achaoui, André Diatta, Muamer Kadic, Sébastien Guenneau

**Affiliations:** 1 Institut FEMTO-ST, UMR 6174, CNRS, Université de Bourgogne Franche-Comté, 25000 Besanco̧n, France; muamer.kadic@kit.edu; 2 Aix Marseille University, CNRS, Centrale Marseille, Institut Fresnel, 13013 Marseille, France; diatta@fresnel.fr (A.D.); sebastien.guenneau@fresnel.fr (S.G.)

**Keywords:** swiss rolls, chiral elastic cloak, Willis coupling, Cosserat medium, elastodynamic cloak, transformation elastodynamics

## Abstract

We propose a design of cylindrical cloak for coupled in-plane shear waves consisting of concentric layers of sub-wavelength resonant stress-free inclusions shaped as Swiss rolls. The scaling factor between inclusions’ sizes is according to Pendry’s transform. Unlike the hitherto known situations, the present geometric transform starts from a Willis medium and further assumes that displacement fields u in original medium and $u^{\prime }$ in transformed medium remain unaffected ($u^{\prime }=u$). This breaks the minor symmetries of the rank-4 and rank-3 tensors in the Willis equation that describe the transformed effective medium. We achieve some cloaking for a shear polarized source at specific, resonant sub-wavelength, frequencies, when it is located in close proximity to a clamped obstacle surrounded by the structured cloak. The structured medium approximating the effective medium allows for strong Willis coupling, notwithstanding potential chiral elastic effects, and thus mitigates roles of Willis and Cosserat media in the achieved elastodynamic cloaking.

## 1. Introduction

Following the work of Milton, Briane and Willis [1], a new field has emerged in metamaterials: transformed elastic media make a region neutral to fully coupled cylindrical [2] and spherical [3] elastic waves. There are various routes to elastic cloaking, which have been listed in [4,5], and we shall focus here on one of these based on the concept of some unconventional effective dynamic properties enabling both minor symmetry breaking in the rank-4 elasticity tensor and non-vanishing rank-3 and 2 tensors in the Willis model [6] near resonant frequencies of certain types of stress-free inclusions shaped as Swiss rolls. Swiss rolls were introduced in the context of electromagnetic metamaterials for artificial chirality by Pendry and co-workers [7], and such magneto-optic coupling recently found a counterpart in elasticity [8,9,10,11].

## 2. Transformed Willis Equations and Minor Symmetry Breaking

We note that the idea of Willis media [6] described in the case of time-harmonic waves by

(1) $$\nabla_{x}\cdot \left(C:\nabla_{x}u+S\cdot u\right)+\underline{\underline{\rho }}\omega^{2}u=-D:\nabla_{x}u\;,$$

with ω the angular wave frequency, the rank-4 elasticity tensor C with all its minor and major symmetries, as well as the rank-3 elasticity tensors S and D such that $D_{pqr}=-S_{qrp}$, and the rank-2 (symmetric) density tensor $\underline{\underline{\rho }}$, was introduced as a promising route to elastodynamic cloaking and as a solution to the non-invariance of the Navier equation under general change of coordinates [1].

This was achieved thanks to a properly chosen gauge linking the displacement fields u and $u^{\prime }$ through the Jacobian of the transformation. As pointed out in [2,3], if one assumes that $u=u^{\prime }$ the time-harmonic Navier equation

(2) $$\nabla_{x}\cdot \left(C:\nabla_{x}u\right)+\rho \omega^{2}u=0\;,$$

retains its form under coordinate change, but the elasticity tensor C loses its minor symmetry. Other choices of the gauge lead to different types of transformed media [4].

In this article, we start from a Willis material, as our background material and transform it into a new material with some specific properties. If we consider the coordinate change $\phi :x=\left(x_{1},x_{2},\cdots \right)⟼x^{\prime }=\left(x_{1}^{\prime }\left(x\right),x_{2}^{\prime }\left(x\right),\cdots \right)$ and we impose that the displacement $u=u^{\prime }$ in Willis’s Equation (1), this equation is actually form invariant, but the tensors therein lose their minor symmetries. This kind of transformed Willis medium therefore encapsulates some features of Cosserat media and the expressions of transformed tensors $C^{\prime }$, $D^{\prime }$, $S^{\prime }$ and ${\underline{\underline{\rho }}}^{\prime }$ that reflect the minor symmetry breaking are given in Appendix A.

## 3. Periodic Medium with Stress-Free Swiss Rolls and Resonances

Our observation of the possibility of a transformed Willis medium with Cosserat-like tensors opens interesting avenues for the design of cylindrical elastodynamic cloaks via homogenization approaches combining recent findings in metamaterials displaying strong Willis coupling [12,13] and chiral elasticity features [8,11], as we shall see in the sequel. To exemplify the usefulness of the transformed Willis equation with non-fully symmetric elasticity tensors, we propose to design a microstructured cloak consisting of Swiss rolls displaying the usual features encountered in both Cosserat and Willis media in the low frequency limit.

Let us first consider a simplified form of Navier equation that governs the propagation of time-harmonic elastic waves in an isotropic homogeneous elastic medium

(3) $$\left(\lambda +2\mu \right)\nabla \nabla \cdot u-\mu \nabla \times \nabla \times u+\rho \omega^{2}u=0$$

where λ and μ are the compressional and shear Lamé coefficients, ρ is the density and ω is the angular wave frequency.

If the homogeneous medium is structured with inclusions, one can supply (3) with boundary conditions, such as clamped u=0 or stress-free σ(u)·n=(C:ϵ(u))·n=0 where C is the rank-4 elasticity tensor with entries $C_{ijkl}=\lambda \delta_{ij}\delta_{kl}+\mu \left(\delta_{ik}\delta_{jl}+\delta_{il}\delta_{jk}\right)$ and σ(u) and ϵ(u) are the rank-2 stress and strain tensors with entries $\sigma_{ij}=\lambda \varepsilon_{kk}\delta_{ij}+2\mu \varepsilon_{ij}$ and $\varepsilon_{ij}=1/2\left(\partial u_{i}/\partial x_{j}+\partial u_{j}/\partial x_{i}\right)$, respectively, n being the outward pointing normal to the boundary of inclusions. It is then easily seen from the Helmholtz decomposition u=∇Φ+∇×Ψ,∇·Ψ=0, with scalar (pressure related) and vector (shear related) Lamé potentials Φ and Ψ that for stress-free inclusion, pressure (p) and shear (s) waves in (3) are now coupled. Indeed, there is a conversion of p in s waves (and vice versa) at any stress-free boundary, and this coupling has been used previously notably for opto-elastic switches in arrays of stress-free holes in silica [14].

**Figure 1:**
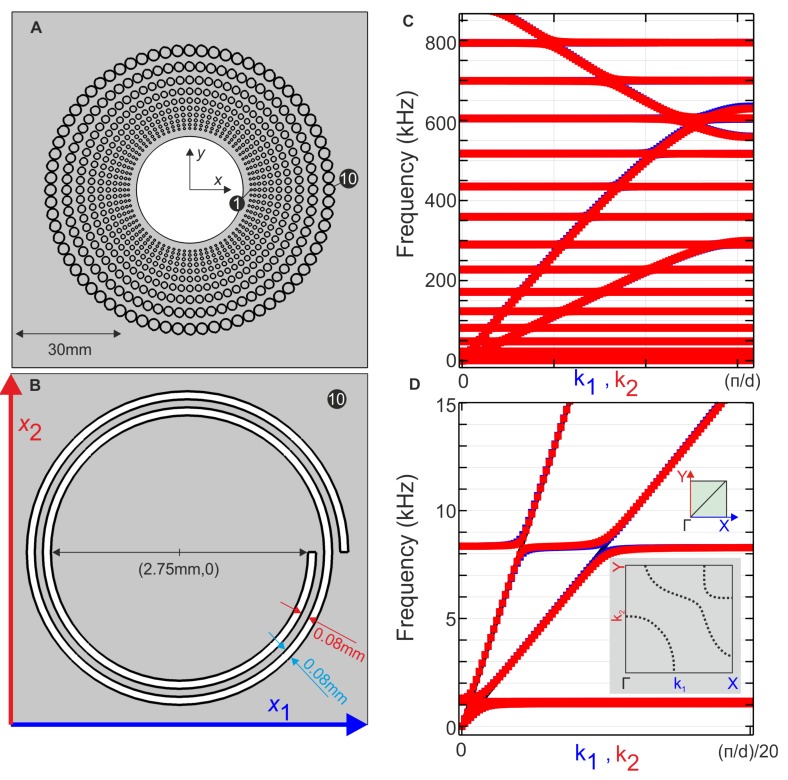
Geometrical characteristics and dispersion properties of the investigated model. (**A**) Geometry of the entire cloak; (**B**) Zoom on an elementary cell; (**C**) Band diagram for a Bloch vector k running along ΓM ($k=(k_{1},0)$ with $k_{1}\in \left[0,\pi /d\right]$) and along MY ($k=(\pi /d,k_{2}$) with $k_{2}\in \left[0,\pi /d\right]$), showing the effective medium is isotropic; (**D**) Zoom-in in the neighborhood of Γ, where one notes the avoided crossings at resonances around 1 kHz and 8 kHz;. These dispersion curves serve as a guide for our homogenized model with an inset showing the isofrequencies around the resonance (approximation of a Willis-type medium).

Let us now assume that a homogeneous medium is structured with a square array of stress-free inclusions shaped as Swiss rolls invariant along the $x_{3}$-axis, as shown in Figure 1. Thanks to this invariance, we can consider in-plane coupled shear and pressure elastic waves on one hand, with unknown $\left(u_{1},u_{2},0\right)$ and anti-plane shear waves with unknown $\left(0,0,u_{3}\right)$ in (3), on the other hand. We focus on the former. The periodicity of the cladding implies that the in-plane displacement field $u=(u_{1},u_{2})$ satisfies the Floquet–Bloch theorem:

(4) $$u\left(x_{1}+d,x_{2}+d\right)=u_{k}\left(x_{1},x_{2}\right)\exp\left(i\left(k_{1}d+k_{2}d\right)\right)$$

where $k=(k_{1},k_{2})$ is the Bloch vector which describes the first Brillouin zone (BZ) ΓMX in the reciprocal space, with Γ=(0,0), M=(π/d,0) and X=(π/d,π/d) and *d* the array pitch. One can then look for eigenfrequencies $\omega_{k}$ and associated Floquet–Bloch eigenfields $u_{k}$ solutions of (3), and by letting k vary within BZ we compute some dispersion diagrams. We display in Figure 1 geometric characteristics of the Swiss rolls under study (panels A, B) and associated dispersion curves along ΓM (green curves) and MX (red curves), see panel C.

One notes that flat bands correspond to localized modes associated with resonances of the Swiss rolls that are reasonably well approximated (within 10% of error margin) by transverse vibrations a corresponding unrolled beam with clamped conditions at one end and stress-free at the other end (see e.g., Chapter 4 in [15]), in the similar way to what was done in [16] in the electromagnetic case. This elementary model could be improved for instance using effective medium approaches detailed in [17,18]. It is also observed in panel D that wavespeed of s waves differs markedly along ΓM and MX directions, which is interpreted as a dynamic anisotropic mass density (i.e. a symmetric rank-2 tensor).

## 4. Effective Properties and Cloaking

Let us now note that properties of the effective symmetric rank-4 elasticity tensor C, rank-3 elasticity tensors S and D such that $D_{pqr}=-S_{qrp}$ and rank-2 density tensor $\underline{\underline{\rho }}$ are inferred from a retrieval method such as what was done in [8,11], or alternatively from a direct Bloch-wave [19] homogenization approach applied to the doubly periodic array of identical Swiss rolls in Cartesian coordinates, see Figure 1. Thus far, the effective medium is a conventional Willis medium.

**Figure 2:**
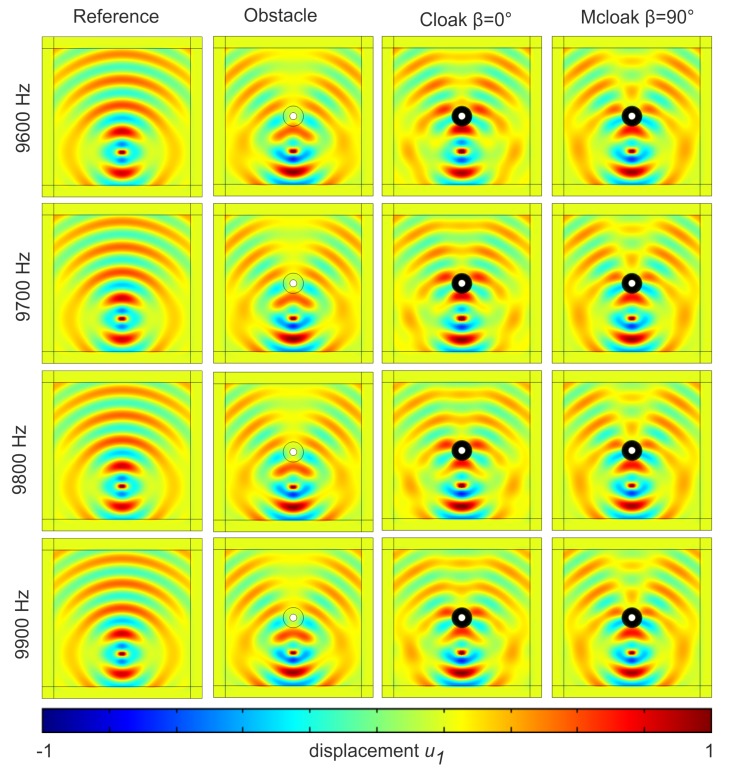
In-plane shear elastic wave generated by a point force located at $\left(x_{1},x_{2}\right)=\left(0,-150\right)$ and oriented along the *x*-axis. This S wave propagates within an isotropic homogeneous elastic bulk (here PMMA) with a cloak centered at (0,0) of inner radius $r_{1}=1.5$ cm and outer radius $r_{2}=4$ cm and consisting of 11 concentric layers of Swiss rolls made of a soft material ($\lambda =601010^{5}$ Pa and $\mu =4.10^{4}$ Pa). The wave frequency ranges from 9.6 to 9.9 kHz. Please note that Cartesian elastic Perfectly Matched Layers have been set on either sides of the square computational domain. First column is for the shear polarized point source in PMMA (benchmark); Second column has a clamped obstacle centered at (0,0) of radius $r_{0}=3$ cm; Third column is for the source with clamped obstacle and cloak. Fourth column is same when the Swiss rolls have been tilted through an angle β=π/2 about their gravity center, which is a modified cloak (Mcloak). Scattering of clamped obstacle is reduced for cloak, unlike for Mcloak.

However, as explained in the previous section, transformation physics affects the Willis Equation (1), although it retains its form if we assume that $u=u^{\prime }$, and so when we apply Pendry’s transform (which maps a disc onto a corona, thus creating a hole known as invisibility region, see [20]) to the doubly periodic array of Swiss rolls, the symmetry of the tensors in the effective Willis equation gets broken, and besides from that tensors become spatially varying. Consequently, the effective Willis equation describing the cloak with gradually varying Swiss rolls in Figure 2 has the form of Equations (7)–(10) in Appendix A.

Therefore, when we map the doubly periodic array of Swiss rolls on a transformed medium using Pendry’s transform, the transformed Willis medium now has built-in Cosserat features (i.e., a minor symmetry breaking in some tensors). The Swiss roll-based cloak is an example that illustrates this type of combined mechanisms, in which cloaking is due to both Willis and Cosserat materials. Indeed, a wave is usually characterized by its polarization, a direction of the wavenumber, a frequency and a rated velocity. The dynamic density can straightforwardly be omitted since we are exciting the propagation at a unique nominal frequency. However, the inertial behavior of the Swiss rolls entails a change in direction of the wave propagation to circumvent the obstacle and is naturally accompanied by mode conversion (each inclusion becomes a secondary source of waves). Mathematically speaking, this involves both the symmetry breaking of the fourth and third order elastic tensors in the Willis-type equation. Our future goal is to rigorously quantify the weighting of each contribution regarding geometrical and physical properties of the Swiss rolls. Interestingly, similar effective parameters for a chiral Willis medium have been deduced from a retrieval method in [10] applied to the mechanical metamaterial first introduced in [8] in the context Eringen equations [21,22], which are the counterpart of bianisotropic equations in optics [23].

## 5. Physical Discussion of Band Diagrams

The magneto-optic coupling is actually easily seen using classical homogenization techniques in [24], and same techniques could be applied to the effective medium description of the Willis coupling for our array of Swiss rolls. In fact, one can alternatively deduce these features from the reading of band diagrams. When a bunch of resonant elements meet the wave propagation, a strong coupling between the so-called continuum and the resonators may occur. This can directly be identified in the band diagrams in Figure 1 through band repealing between a polarized continuum and the flat mode describing the energy trapping in the resonator. This level repulsion can reach its maximum with the appearance of band gaps. The latter describes the energy prohibition inside the periodic structure through a total reflection, energy storage or conversion to other types of modes. A straight crossing between the bands reveals no interaction between the resonators and the continuum as has been reported in [25]. In the latter paper, we have studied the possibility of a resonator to drastically change the direction of the wave for focusing purposes. In Figure 1A, we show a sketch of the Swiss-roll-based cloak with a zoom inset in Figure 1B. In Figure 1C, we depict the normalized band structure of a periodic structure made of inclusions shaped in Swiss-roll resonators (Figure 1B). This band diagram shows mainly the two modes longitudinal and transverse starting from Γ point and tremendous flat bands describing the resonance frequencies of the inclusion. It is worth noting that the number and the position of these bands in a given frequency range depend directly on the length of the spiral constituting the Swiss rolls, as can be inferred from our simple asymptotic model of unrolled Swiss rolls borrowed from [16]. Hence, the cloak has been conceived in a way that most of the resonances are gathered in a tiny range of frequency. This choice was made to optimize the functionality of the inclusions while rolling. A zoom-in on the band structure near a resonance frequency is illustrated in Figure 1D. We can clearly observe that the flat band and the continuum repeal slightly from each other without creating a bandgap. Though the inclusion shaped as a Swiss-roll is a bad candidate to achieve perfect reflectors, at this stage we are confident that this weak coupling to the continuum added to the potential of the inclusion to rotate under an incoming wave would contribute drastically to deflect the wave propagation. Furthermore, the level repulsion band anti-crossing between the flat mode and the continuum depends on the direction of the propagation just as well as the inclusion orientation (Figure 1D). To illustrate more this more or less strong coupling, we computed the isofrequency contours. In the inset of Figure 1D, the latter were evaluated around a frequency resonance. To be more consistent, let us split the Brillouin Zone into two subsurfaces; i.e., GXM and GYM. Three bands (p, s and coupled p-s) are identified around the frequency 8 kHz and each one is extended barely the same way in the two subsurfaces. If we look more closely we can notice that two kinds of anisotropy can be observed. The first one is the position of the wavenumbers. We can remark that for both p and s polarizations, the wave velocity toward GX is slightly fast compared to GY. The second anisotropy concerns the wave trapping (or coupling between the continuum and the resonator). We stress here that this coupling depends not on the wavevector but on the polarization of the wave (note the line width of the curves).

**Figure 3:**
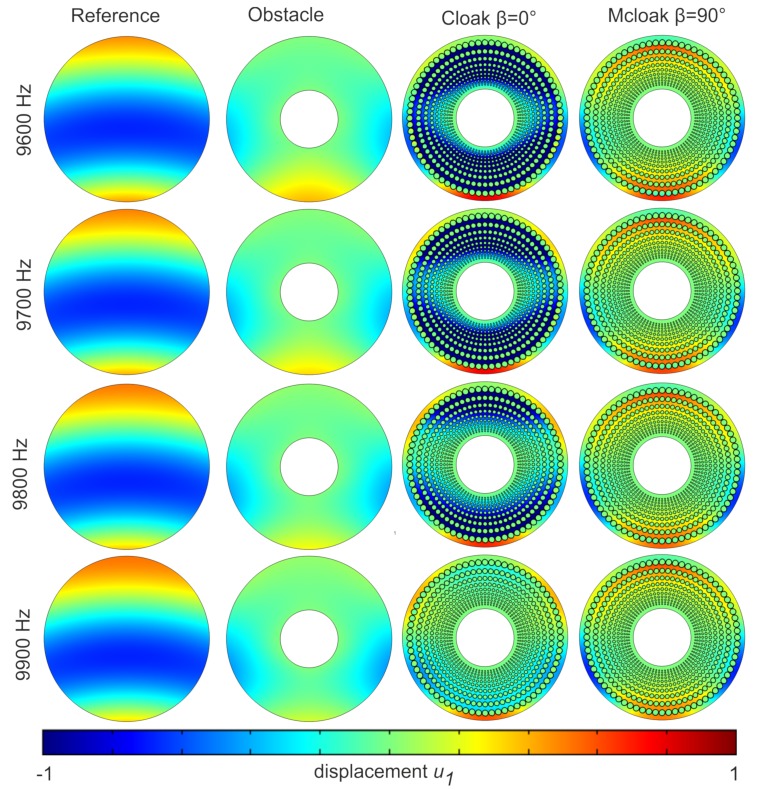
Field plots as in Figure 2 but shown only around the cloak’s region.

We test our cloak in Figure 2 near resonances of the Swiss rolls, which have been scaled up and down with respect to Figure 1, depending upon whether they are located on outer or inner, rings of the cloak in Figure 2. We consider the frequency range from 9.6 to 9.9 kHz and pick up some resonant frequencies of some Swiss rolls. Upon inspection of the case of a shear polarized point source in homogeneous medium (first row), same source in the presence of a clamped obstacle without cloak (second row), with cloak (third row) and with a cloak without the proper design (fourth row), we deduce that cloaking is achieved i.e., the magnitude of the shear wave is recovered in forward scattering in third column, although with a slight phase delay induced by the longer wave trajectory induced by the cloak design. To exemplify the mechanism of the cloak, we show a magnified view of these plots in Figure 3.

## 6. Conclusions

In this article, we have proposed to approximate a Willis-type elastodynamic cloak with an elastic isotropic medium structured with stress-free Swiss rolls of gradually varying sizes, according to Pendry’s transform. We have considered a coordinate change ϕ such that $u^{\prime }\left(x\right)=u\left(x\right)$, in which case the transformed Willis equation has the exact same structure as (1), but with a transformed elasticity tensor $C^{\prime }$ without the minor symmetries and same for the rank-3 tensors. Note however that the density could be a scalar, and in any case it is fully symmetric. The cloak we have designed is thus neither totally of the Willis-type [1], nor totally of the Cosserat type [2,3]. Finally, we note the alternative route of direct lattice transforms [26,27,28] towards elastodynamic cloaking, which does not make use of resonant structural elements and thus follows a different protocol. In the near future, we would like to compare numerically and experimentally the efficiency of our cloak’s design with those in [27,28] in various scenarios. Finally, while completing the revised version of the manuscript, we have been made aware of a related work on a Cosserat cloak [29].

## Appendix A. On Willis Medium with Cosserat Coefficients

We start from a Willis material with elasticity order 4 tensors $C^{w},$ order 3 tensors $S^{w}$, $D^{w}$ and mass density $\rho^{w}$ satisfying the Willis Equations

(A1) $$\begin{array}{ccc} 0 & = & \nabla_{x}\cdot \left(C^{w}:\nabla_{x}u+S^{w}\cdot u\right)\\ \; & \; & +D^{w}:\nabla_{x}u+\omega^{2}\rho^{w}u \end{array}$$

We apply a transformation $x\mapsto x^{\prime }$ with Jacobian matrix J by requiring that the original and transformed displacements be the same. Please note that this is the requirement applied to the transformation scheme that produces Cosserat material, starting from a background material which is isotropic homogeneous [2,3]. In this appendix, we pioneer a new scheme consisting on applying this transformation technique to a Willis material, as our background reference material. As one can see below, the resulting transformed material will satisfy the same equations as Willis equations, but will however have elasticity tensor, hereafter denoted by $C^{cw}$, which no longer possesses minor symmetries. The resulting tensors $S^{cw}$ and $D^{cw}$ of order 3, still satisfy the identities $S_{ijk}^{cw}=-D_{kij}^{cw}$, but now the coefficients $S_{ijk}^{cw}$ and $S_{jik}^{cw}$ are generally not equal.

We apply a (general) transformation $x=\left(x_{1},x_{2},\cdots \right)⟼x^{\prime }=\left(x_{1}^{\prime }\left(x\right),x_{2}^{\prime }\left(x\right),\cdots \right)$ mapping $\Omega \subset R^{n}$ to $\Omega^{\prime }\subset R^{n}$ and we further impose that the displacement $u^{\prime }$ in the transformed material filling $\Omega^{\prime }$, be the same as the displacement u in the background material (untransformed original material) in Ω. We use the notations ${\left(\nabla_{x}u\right)}_{ij}=\partial u_{j}/\partial x_{i}$, ${\left(\nabla_{x^{\prime }}u\right)}_{ij}=\partial u_{j}/\partial x_{i}^{\prime }$ and $J^{T}$ stands for the transpose of the Jacobian J, with $J_{ij}=\partial x_{i}^{\prime }/\partial x_{j}$. We next use the weak formulation of (1): for any test function ϕ,

(A2) $$\begin{array}{ccc} 0 & = & \int_{\Omega }[\nabla_{x}\cdot \left(C^{w}:\nabla_{x}u+S^{w}\cdot u\right)\\ \; & + & D^{w}:\nabla_{x}u+\rho^{w}\omega^{2}u]\cdot \phi \;dx\\ \; & = & \int_{\Omega }[-\left(C^{w}:\nabla_{x}u+S^{w}\cdot u\right):\nabla_{x}\phi \\ \; & + & \left(D^{w}:\nabla_{x}u+\rho^{w}\omega^{2}u\right)\cdot \phi ]dx\\ \; & = & \int_{\Omega^{\prime }}[-\left(C^{w}:J^{T}\nabla_{x^{\prime }}\;u+S^{w}\cdot \;u\right):J^{T}\;\nabla_{x^{\prime }}\;\phi \\ \; & + & \;\;\left(D^{w}:J^{T}\nabla_{x^{\prime }}u+\rho^{w}\omega^{2}u\right)\cdot Œ]\;\det\left(J^{-1}\right)\;dx^{\prime } \end{array}$$

Simply further developing the above we deduce the following

(A3) $$\begin{array}{ccc} 0 & = & \nabla_{x^{\prime }}.\left[C^{cw}:\nabla_{x^{\prime }}u+S^{cw}\cdot u\right]\\ \; & \; & +D^{cw}:\nabla_{x^{\prime }}u+\omega^{2}\rho^{cw}u \end{array}$$

with

(A4) $$\begin{array}{ccc} C_{ijkl}^{cw} & = & \frac{1}{\det(J)}\sum_{p,q}\frac{\partial x_{i}^{\prime }}{\partial x_{p}}\frac{\partial x_{k}^{\prime }}{\partial x_{q}}C_{pjql}^{w},\\ S_{ijk}^{cw} & = & \frac{1}{\det(J)}\sum_{s}\frac{\partial x_{i}^{\prime }}{\partial x_{s}}S_{sjk}^{w},\\ D_{ijk}^{cw} & = & \frac{1}{\det(J)}\sum_{s}\frac{\partial x_{j}^{\prime }}{\partial x_{s}}D_{isk}^{w}=-S_{jki}^{cw},\\ \rho^{cw} & = & \frac{1}{\det(J)}\rho^{w}. \end{array}$$

If we apply to the above the radial transformation $\left(r,\theta \right)\mapsto \left(r^{\prime },\theta^{\prime }\right):=\left(\frac{r_{2}-r_{1}}{r_{2}}r+r_{1},\;\theta \right)$ in polar coordinates, then the only non-vanishing coefficients of the Cosserat-Willis tensor $C^{cw}$ are

(A5) $$\begin{array}{ccc} C_{r^{\prime }r^{\prime }r^{\prime }r^{\prime }}^{cw} & = & \frac{r^{\prime }-r_{1}}{r^{\prime }}C_{r^{\prime }r^{\prime }r^{\prime }r^{\prime }}^{w},\;C_{r^{\prime }r^{\prime }\theta \theta }^{cw}=C_{r^{\prime }r^{\prime }\theta \theta }^{w},\\ C_{r^{\prime }\theta r^{\prime }\theta }^{cw} & = & \frac{r^{\prime }-r_{1}}{r^{\prime }}C_{r^{\prime }\theta r^{\prime }\theta }^{w},\;C_{r^{\prime }\theta \theta r^{\prime }}^{cw}=C_{r^{\prime }\theta \theta r^{\prime }}^{w},\\ C_{\theta r^{\prime }r^{\prime }\theta }^{cw} & = & C_{\theta r^{\prime }r^{\prime }\theta }^{w},\;C_{\theta r^{\prime }\theta r^{\prime }}^{cw}=\frac{r^{\prime }}{r^{\prime }-r1}C_{\theta r^{\prime }\theta r^{\prime }}^{w},\\ \;C_{\theta \theta r^{\prime }r^{\prime }}^{cw} & = & C_{\theta \theta r^{\prime }r^{\prime }}^{w},\;C_{\theta \theta \theta \theta }^{cw}=\frac{r^{\prime }}{r^{\prime }-r_{1}}C_{\theta \theta \theta \theta }^{w}.\\ S_{r^{\prime }jk}^{cw} & = & \frac{r_{2}}{r_{2}-r_{1}}\frac{r^{\prime }-r_{1}}{r^{\prime }}S_{r^{\prime }jk}^{w}=-D_{kr^{\prime }j}^{cw},\;j,k=r^{\prime },\theta ,\\ S_{\theta jk}^{cw} & = & \frac{r_{2}}{r_{2}-r_{1}}S_{\theta jk}^{w}=-D_{k\theta j}^{cw},\;j,k=r^{\prime },\theta^{\prime }. \end{array}$$

and the Cosserat-Willis density is

(A6) $$\rho^{cw}=\frac{r_{2}^{2}}{{\left(r_{2}-r_{1}\right)}^{2}}\frac{r^{\prime }-r_{1}}{r^{\prime }}\rho^{w}.$$
